# A method of crack detection based on digital image correlation for simulated cracked tooth

**DOI:** 10.1186/s12903-021-01897-2

**Published:** 2021-10-19

**Authors:** Chunliang Zhang, Diwei Mo, Juncheng Guo, Wenlong Wang, Shangbin Long, Houyao Zhu, Danying Chen, Guanghua Ge, Yadong Tang

**Affiliations:** 1 School of Mechanical and Electrical Engineering, Guangzhou University, Guangzhou, 510006 China; 2 Hospital of Stomatology, Sun Yat-Sen University, Guangdong Provincial Key Laboratory of Stomatology, Guangzhou, 510006 China; 3 Department of Dentistry, Hospital of Guangdong University of Technology, Guangdong University of Technology, Guangzhou, 510006 China; 4 School of Biomedical and Pharmaceutical Sciences, Guangdong University of Technology, Guangzhou, 510006 China

**Keywords:** Imaging diagnosis, Cracked tooth, Non-contact measurement

## Abstract

**Background:**

Early clinical cracked tooth can be a perplexing disorder to diagnose and manage. One of the key problems for the diagnosis of the cracked tooth is the detection of the location of the surface crack.

**Methods:**

This paper proposes an image-based method for the detection of the micro-crack in the simulated cracked tooth. A homemade three-axis motion platform mounted with a telecentric lens was built as an image acquisition system to observe the surface of the simulated cracked tooth, which was under compression with a magnitude of the masticatory force. By using digital image correlation (DIC), the deformation map for the crown surface of the cracked tooth was calculated. Through image analysis, the micro-crack was quantitatively visualized and characterized.

**Results:**

The skeleton of the crack path was successfully extracted from the image of the principal strain field, which was further verified by the image from micro-CT. Based on crack kinematics, the crack opening displacement was quantitatively calculated to be 2–10 µm under the normal mastication stress, which was in good agreement with the value reported in the literature.

**Conclusions:**

The crack on the surface of the simulated cracked tooth could be detected based on the proposed DIC-based method. The proposed method may provide a new solution for the rapid clinical diagnosis of cracked teeth and the calculated crack information would be helpful for the subsequent clinical treatment of cracked teeth.

**Supplementary Information:**

The online version contains supplementary material available at 10.1186/s12903-021-01897-2.

## Introduction

Cracked tooth syndrome, also known as incomplete tooth crack, was defined as non-physiological microcracks that normally occur on the surface of the tooth crown [1]. The disease is very easy to be misdiagnosed or mistreated due to the indiscoverable microcracks and the inconspicuous early clinical symptoms [2]. If the cracked tooth is not treated properly, the cracks would continue to expand and eventually leading to the onset of pulpitis or the fracture of the whole tooth [3]. The main objective for the diagnosis of the cracked tooth was to detect the micro-cracks in the early stage of the disease. In general, the clinical diagnosis methods mainly included visual inspection [4], periodontal probing [5], bite diagnosis [6], pulp vitality test [7], staining [8], transillumination [9], computed tomography (CT) [10], etc. More recently, the method like optical coherence tomography (OCT) used for surface crack detection was widely studied [11, 12]. It should be noted that compared with traditional clinical dental imaging techniques, micro-CT and especially cone-beam computed tomography (CBCT) seemed to be effective in detecting longitudinal tooth cracks [13, 14].

However, the available diagnosis methods may have some limitations. Methods like visual inspection, staining, bite diagnosis, etc., may somehow depend on the experience of the clinicians. Although CBCT had a good imaging resolution, the associated radiation would be undesirable. Some non-destructive engineering testing such as ultrasound [15], surface laser [16] may have side effects like pain or discomfort. The complex interactive mechanism between the stimuli signal and the irregular tooth structure may also bring some difficulties for imaging and signal treatment in further clinical diagnosis.

Image-based method implemented with artificial intelligence algorithms [17–19] was also widely performed for crack detection in industry non-destructive testing for the purpose of automation. Unfortunately, the accuracy of such methods may strongly depend on the amount of the training data, which was impractical for the clinical crack tooth diagnosis. Image-based indirect crack detection method was mainly based on its deformation field which was calculated by digital image correlation (DIC). DIC had main advantages like non-contact, simple equipment and high accuracy [20]. Its main idea was to obtain the displacement or deformation of the image by comparing the reference image with the deformed image, which was conducted by the correlation calculation according to the image grey intensity before and after the deformation [21]. Many applications of crack detection were developed by DIC. For example, Landon Dellenbaugh et al. [22] proposed a method for the characterization of the distortion-induced fatigue crack by DIC. Navid Hasheminejad et al. [23] used the correlation of digital images to estimate the strain field on the surface of the asphalt sample. They predicted the locations of cracks to study the healing phenomenon of asphalt concrete. Nishikawa et al. [24] used DIC technology to establish an automatic in situ fatigue observation system to monitor the behavior of small fatigue cracks on the microstructure. Recently, Gehri et al. [31] proposed a DIC-based method to achieve automated crack detection in the field of engineering structure monitoring. In their work, the complex crack pattern could be extracted, and more importantly, quantitative characterization of the crack like the crack opening displacement could also be visualized based on crack kinematics. Although some researches can be found related to stress or strain response of the dental implant through DIC analysis, the detection of the cracked tooth using DIC-based method has not been reported according to our best knowledge.

In this article, we firstly proposed a method based on DIC that was used for crack detection of the simulated cracked tooth. The crack opening displacement was quantitatively visualized. And the systematic random error was measured. The method provided here may be helpful for further development of portable nondestructive testing equipment for clinical detection of oral cracked teeth.

## Methods

### Simulated cracked tooth preparation

The ideal research subject is the fresh extracted cracked tooth, however, which is much more difficult to obtain intactly. Therefore, a reasonable in vitro simulated cracked tooth is necessary. It should be noted that the sample used in this work is not the fresh extracted cracked tooth. The “simulated” here is meant to signify that the crack in the tooth was generated artificially. In this study, the simulated cracked tooth was created based on the phenomena of thermal expansion and contraction. Due to different coefficients of thermal expansion of dental enamel and dentin (two main components of a tooth) [25], damage or crack could be made both on the surface of the tooth crown and in the body of the tooth, if the temperature changing process was carefully controlled. The simulated cracked tooth in vitro seems to fit the definition of cracked tooth syndrome, in which the affected tooth possesses incomplete fracture. Meanwhile, the simulated in vitro sample may induce internal crack contrast to the external mechanical crack-generating methods [8, 26, 27].

Intact extracted human third molar was used in the experiment. After surface cleaning, the sample was put into liquid nitrogen for 10–24 h, and then it was transferred to warm water with the temperature of 60–90 °C for 5–10 min. The large temperature difference generated thermal stress in the sample, which further created the micro-cracks in the sample. The cracks of the sample were checked by the optical microscope. And the storage time in water and liquid nitrogen was tested and controlled so that the generated micro-cracks were as tiny as possible. Then, the simulated cracked tooth was sealed with epoxy resin. The surface of the encapsulated cracked tooth sample was grinded and polished until the tooth crown plane was clearly observed.

In order to enhance the image contrast, the polished surface was sprayed with ink speckle by an airbrush (nozzle diameter: 0.2 mm). Uniform distribution of the painted speckle with a diameter of 3–5 µm was achieved by controlling the spraying parameters, such as pressure, distance, and duration.

### Image acquisition

**Fig. 1:**
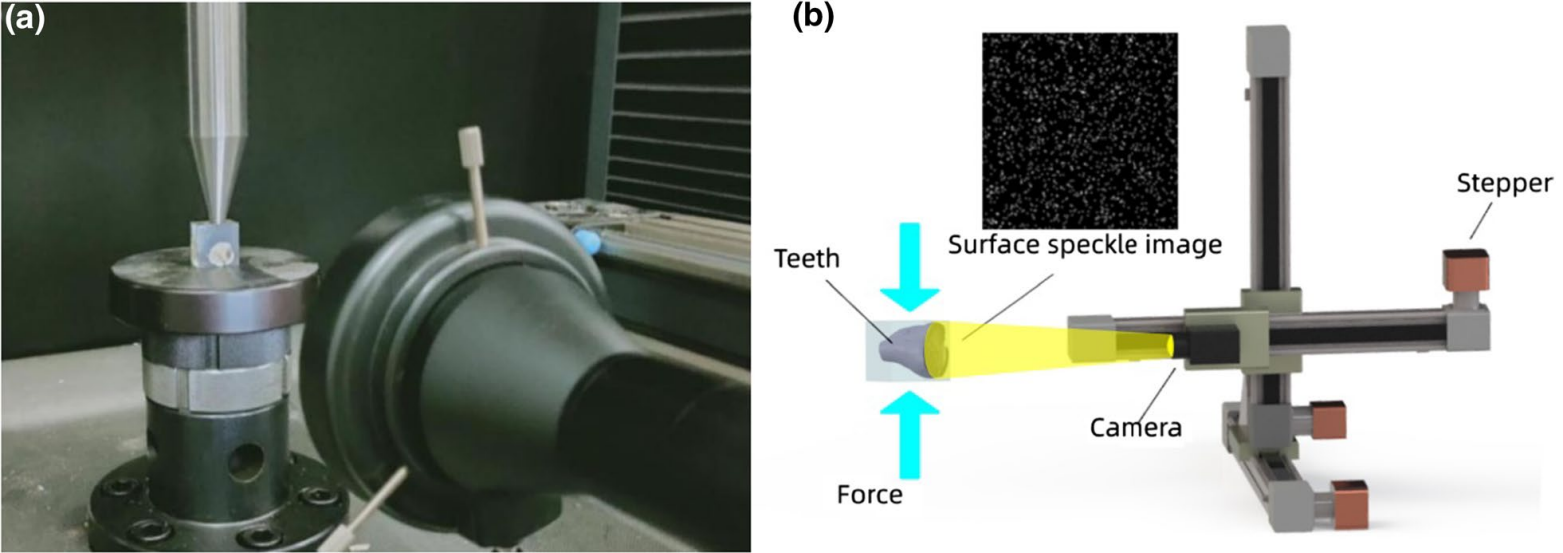
**a** Photo of the setup. **b** Illustration of the experimental setup: cracked tooth sample was sealed in the resin; the compression load was applied to the sample by universal material testing machine; a CCD camera equipped with a telecentric lens was used for image acquisition, which was installed onto a three-axis motion platform

As illustrated in Fig. 1a and b, the simulated cracked tooth sealed with resin was loaded on the universal testing machine which applied compression load onto the sample. The load used here was carefully controlled less than the stress of the normal mastication process. The ink-spayed surface of the simulated cracked tooth crown was observed by a homemade image acquisition setup. The image acquisition setup was mainly constructed with three components: a three-axis motion platform driven by synchronous belts, a CCD camera with white light illuminating system, and a motion-control & image processing system. Each axis of the motion platform was controlled by one stepper motor, which was driven using pulse width modulation through receiving the motion signal from a micro-controller (Uno, Arduino). The upper computer interface was developed based on LabVIEW (National Instruments Technology Co., Ltd, USA), which is responsible for the communication (with micro-controller and CCD camera) and the digital image processing. The CCD camera (MV-SUA2000C/M-T, MindVision Technology Co., Ltd.) used in the study has a resolution of 1920 pixel × 1080 pixel equipped with a telecentric lens (WTL150-04X20, Dehong Vision Technology Co., Ltd. Shenzhen, China) which has a magnification of 0.4×, and the working distance of 150 mm. The distortion induced by the lens was measured less than 0.1%. A supplementary light device was added coaxially in order to have sufficient illumination.

The calibration about the acquisition of the images was achieved automatically by our measuring system. Before the experiment, the system was programmed to acquire a serial of images by changing working distance and the ration angle between the image plane and the object plane. By maximizing the contrast and sharpness of the acquired images, the system was therefore initialized.

### Image processing and strain field calculation

According to the size of the cracked tooth sample, the region of interest (ROI) was selected as 1168 pixels × 876 pixels with the dimension of 22 mm × 16.5 mm. The images (1 pixel approximately represents 20 micro-meters) were then used for DIC calculation. The size of the subzone in the deformed image was selected as 39 pixels × 39 pixels. The searching region in the undeformed image was set as 69 pixels × 69 pixels with a matching step size of 4 pixels, which lead to one minute for one image matching process by a computer equipped with Intel(R) Core (TM)i5-8400 CPU @ 2.80Ghz and 8 GB RAM. Based on the principle of the digital image correlation method, the image processing program compared the reference speckle image with the deformed speckle image and obtained the displacement or deformation filed through correlation calculation according to the given speckle intensity before and after the deformation. The image matching procedure was implemented by the machine vision toolbox of LabVIEW.

A real-time oral diagnostic method for cracked tooth may be preferable. Considering the matching time of the reference and target subsets, in this preliminary study, normalized cross correlation (NCC) [28] was used to calculate the correlation coefficient, which can be described as:

1 $$C(x,y) = \frac{{\sum\nolimits_{y^{\prime} = 0}^{h - 1} {\sum\nolimits_{x^{\prime} = 0}^{w - 1} {{\varvec{T}}(x^{\prime } ,y^{\prime } ){\varvec{I}}(x + x^{\prime } ,y + y^{\prime } )} } }}{{\sqrt {\sum\nolimits_{y^{\prime} = 0}^{h - 1} {\sum\nolimits_{x^{\prime} = 0}^{w - 1} {{\varvec{T}}(x^{\prime } ,y^{\prime } )^{2} \sum\nolimits_{y^{\prime} = 0}^{h - 1} {\sum\nolimits_{x^{\prime} = 0}^{w - 1} {{\varvec{I}}(x + x^{\prime } ,y + y^{\prime } )^{2} } } } } } }}$$

where *C(x, y)* is the calculated correlation coefficient with the value between 0 and 1 (1 means perfect match); $(x^{\prime},y^{\prime})$ are the coordinates of the reference template ${\varvec{T}}$;$(x,y)$ are the coordinates of the deformed image ${\varvec{I}}$; *h* and *w* are the height and width of the template.

A coarse–fine search matching algorithm, namely low-difference sequence sampling image matching method was selected in the matching process, whose details can be found in reference [29]. Once subset area was matched by maximizing the correlation coefficient in the searching area (defined by the step size and the subset size), the displacement field was calculated according to the difference of the location, which can be described as:

2 $${\varvec{u}} = x - x^{\prime }$$

3 $${\varvec{v}} = y - y^{\prime }$$

where ***u*** is the displacement vector in horizontal direction and ***v ***is the displacement in vertical direction. Subpixel accuracy was achieved by grey level interpolation. Considering the processing time, bilinear interpolation was implemented in our case here.

After displacement field was obtained, the resulting image were firstly treated by a median filter in order to decrease the noise. Then the deformation field was calculated [30]. Generally, the discrete displacement data was approximated by a two-dimensional first-order polynomial with ***u*** in x direction and ***v ***in y direction. And the deformation was obtained by calculating the partial derivative of the displacement with respect to its coordinates. The strain can be calculated as [31, 32]:

4 $$\left[ \begin{gathered} a_{0} \hfill \\ a_{1} \hfill \\ a_{2} \hfill \\ \end{gathered} \right] = ({\varvec{P}}^{T} {\varvec{P}})^{ - 1} {\varvec{P}}^{T} {\varvec{u}}$$

5 $$\left[ \begin{gathered} b_{0} \hfill \\ b_{1} \hfill \\ b_{2} \hfill \\ \end{gathered} \right] = ({\varvec{P}}^{T} {\varvec{P}})^{ - 1} {\varvec{P}}^{T} {\varvec{v}}$$

6 $${{\varepsilon}}_{x} = \frac{{\partial {{u}}}}{\partial x} = {{a}}_{1} ,$$

7 $${{\varepsilon}}_{y} = \frac{{\partial {{v}}}}{\partial y} = {{b}}_{2} ,$$

8 $${{\gamma}}_{xy} = \frac{{\partial {{u}}}}{\partial y} + \frac{{\partial {{v}}}}{\partial x} = {{a}}_{2} + {{b}}_{1}$$

where, *a*_0_, *a*_1_, *a*_2_, *b*_0_, *b*_1_, *b*_2_ is the undetermined coefficient, ***(P***^***T***^***P)***^***−1***^***P***^***T***^ is the pseudo inverse of ***P***, ***P ***is the local coordinate matrix, ***u*** is the displacement in x direction, ${\varvec{v}}$ is the displacement in y direction, **ε**_**x**_ is the strain in x direction, **ε**_**y**_ is the strain in y direction, and γ_**xy**_ is the shear strain.

The principal strain can be calculated as [33]:

9 $$\left. \begin{gathered} {\varvec{\varepsilon}}_{1} = {\varvec{\varepsilon}}_{\max } \hfill \\ {\varvec{\varepsilon}}_{2} = {\varvec{\varepsilon}}_{\min } \hfill \\ \end{gathered} \right\} = \frac{{{\varvec{\varepsilon}}_{x} + {\varvec{\varepsilon}}_{y} }}{2} \pm \sqrt {\left( {\frac{{{\varvec{\varepsilon}}_{x} - {\varvec{\varepsilon}}_{y} }}{2}} \right)^{2} + \left( {\frac{{{\varvec{\gamma}}_{xy} }}{2}} \right)^{2} }$$

where ${{\varvec{\upvarepsilon}}}_{\mathbf{m}\mathbf{a}\mathbf{x}}$ is the maximum principal strain, ${{\varvec{\upvarepsilon}}}_{\mathbf{m}\mathbf{i}\mathbf{n}}$ is the minimum principal strain.

Considering the randomly distributed crack on the surface of the simulated cracked tooth, principal strain should be a suitable choice for the further crack skeletonization, because unidirectional strain field (${{\varvec{\upvarepsilon}}}_{\mathbf{x}}$ or ${{\varvec{\upvarepsilon}}}_{\mathbf{y}}$) may lose some information while the crack orientation was parallel to the force direction.

### Crack extraction and analysis

**Fig. 2:**
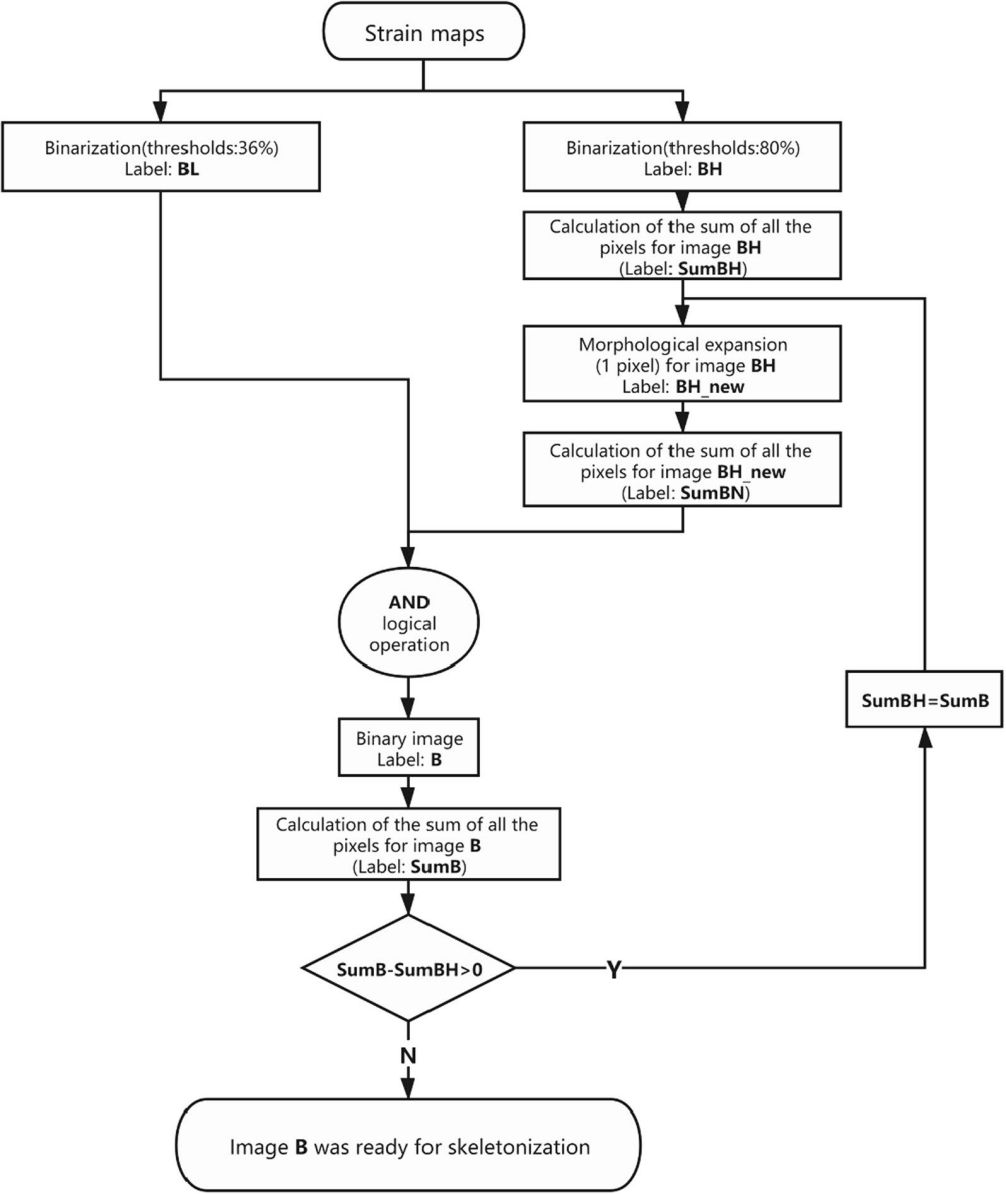
Flowchart of the image treatment process

The skeleton of the crack was extracted through an image treatment process implemented in Matlab. The generated image of the deformation field was treated through the process of binarization, morphological operation and final skeletonization. Single thresholding value may lead to image binarization mixing with noise (branches along with the main crack), which make the skeletonization of the main crack impossible. Here, an iteration process was used to obtain the main crack path by continuously expanding the large strain area (corresponding to the main crack area). The process can be described as a flow chart in Fig. 2. The values of the threshold were well selected after several image treatment experiments.

### Calculation of crack opening displacement

After the crack path was extracted, the crack opening displacement (COD) was quantitatively calculated based on the kinematics of the crack, and the details of the calculation could be found in the work of Gehri et al. [31]. Generally, the crack was considered to be composed of two faces (${\varvec{A}}$ and ${\varvec{B}}$). The relative displacement (${\varvec{\delta}}$) of the two faces of the crack can be calculated as:

10 $${\varvec{\delta}} = {\varvec{\delta}}_{B} - {\varvec{\delta}}_{A}$$

11 $${\varvec{\delta}}_{A} = {\varvec{\delta}}_{{A_{1} }} + \left( {{\varvec{I}}_{2} - {\varvec{R}}_{A} } \right){\varvec{a}}_{1}$$

12 $${\varvec{\delta}}_{B} = {\varvec{\delta}}_{{B_{1} }} + \left( {{\varvec{I}}_{2} - {\varvec{R}}_{B} } \right){\varvec{b}}_{1}$$

where ${{\varvec{R}}}_{{\varvec{A}}}$ and ${{\varvec{R}}}_{{\varvec{B}}}$ are the transformation matrix consisted of the rotation angle for each face.${{\varvec{\delta}}}_{{\varvec{A}}1}$ and ${{\varvec{\delta}}}_{{\varvec{B}}1}$ are crack lip displacement vectors, ${{\varvec{a}}}_{1}$ and ${{\varvec{b}}}_{1}$ are position vectors in the undeformed state.

Based on the crack inclination angle, the calculated displacement ${\varvec{\delta}}$ was decomposed into COD and crack slip displacement. The COD (${\varvec{\delta}}$_***n***_) was then defined as:

13 $${\varvec{\delta}}_{n} = \sin (\alpha_{r} )\left\| {\varvec{\delta}} \right\|$$

where ${\alpha }_{r}$ is the crack inclination angle which would be influenced by the selection of the calculated window size $w$. The parameters used in the calculation were also illustrated in Additional file 1: Fig. S1. In our work here, window size was selected as 7 pixels considering both the calculation cost and imaging resolution.

### Computed tomography

In order to verify the proposed image-based detection method, micro-computed tomography (µCT) was tested for comparison. The simulated cracked tooth was observed by a micro-CT (SkyScan1276, BRUKER, at Guangzhou FocusPower Company). The scanning parameters were set as 20 W, 100 kV, 0.3 µA, ×1 magnification, resulting in an image of 120,912,091,344 × voxels with a voxel size of 20 µm × 20 µm × 20 µm.

The micro-CT image was then treated by adaptive histogram filter and median filter to decrease the noise. Then, the sharpening of the image was made by Laplacian filter to further enhance the feather of the image. After then, the skeletonization of the image was implemented by the function provided in Matlab.

## Results and discussion

### Imaging of the crack location

**Fig. 3:**
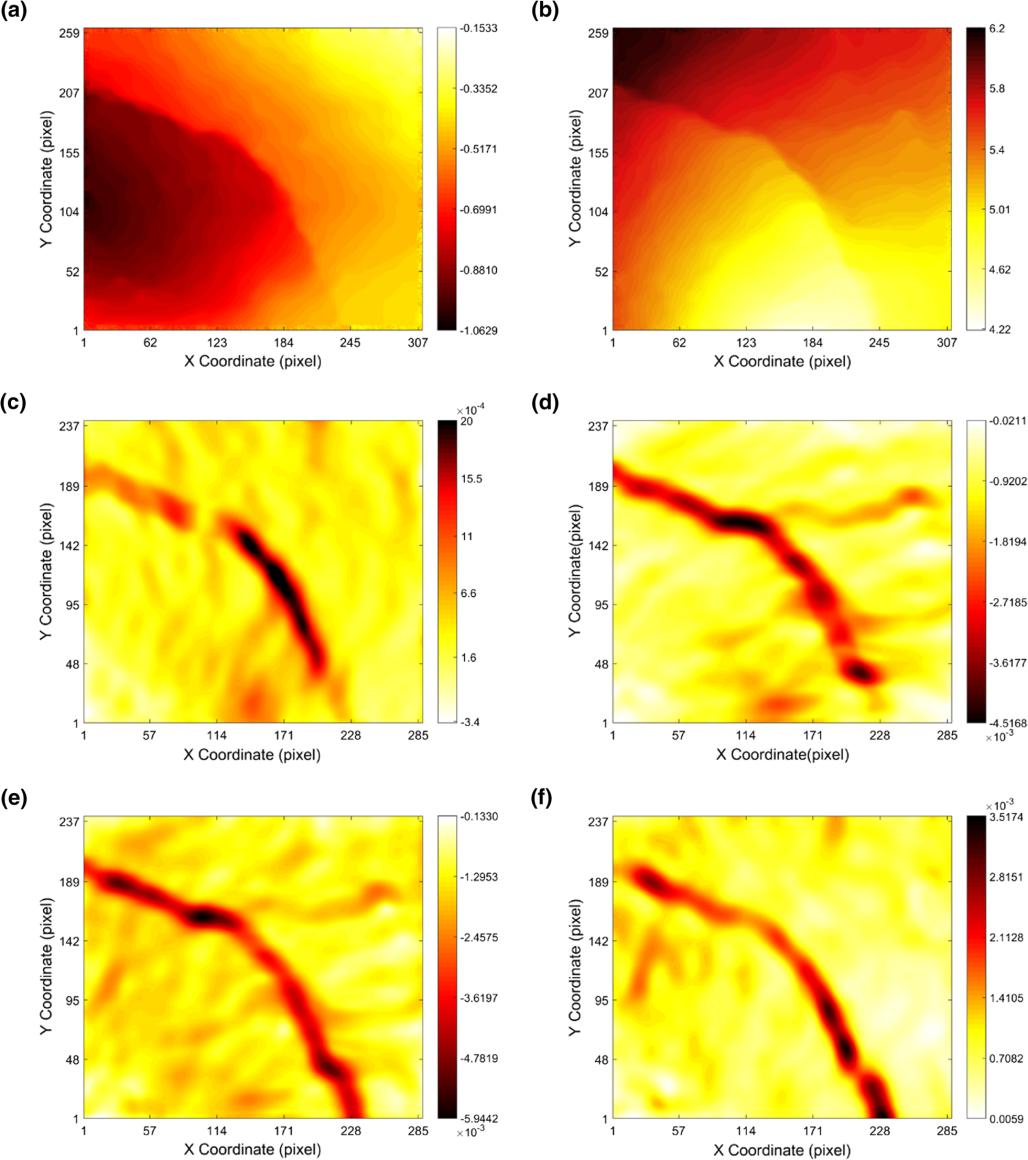
Results of calculated displacement/strain field: **a** displacement in X direction; **b** displacement in Y direction; **c** **ε**_**xx**_ strain; **d** **ε**_**yy**_ strain; **e** minimum principal strain; **f** maximum principal strain. The image here was a cropped area from the resulted DIC map, which further emphasized the region of the crack

As shown in Fig. 3a–d, the calculated displacement field and the strain field in both X and Y directions were plotted. The maximum displacement in each direction was calculated as 1 pixel and 6.2 pixels, which represented 20 µm and 124 µm, respectively (in this system, 1 pixel approximately represents 20 µm). The displacement in Y direction was larger compared to the value in X direction, because the compression force was applied along the Y direction. The tiny displacement value in X direction may imply the slippage of the crack during the compression, which could also be found in reference [34]. The path of the crack could be approximately detected both on X and Y displacement field (or the strain field). However, it could not be considered for further crack localization and skeletonization, because the randomly oriented crack may lead to information loss when the displacement field was only captured for the selected X and Y directions. Thus, a principal strain field may be a better choice for random crack detection, which was also used in the reference [35].

**Fig. 4:**
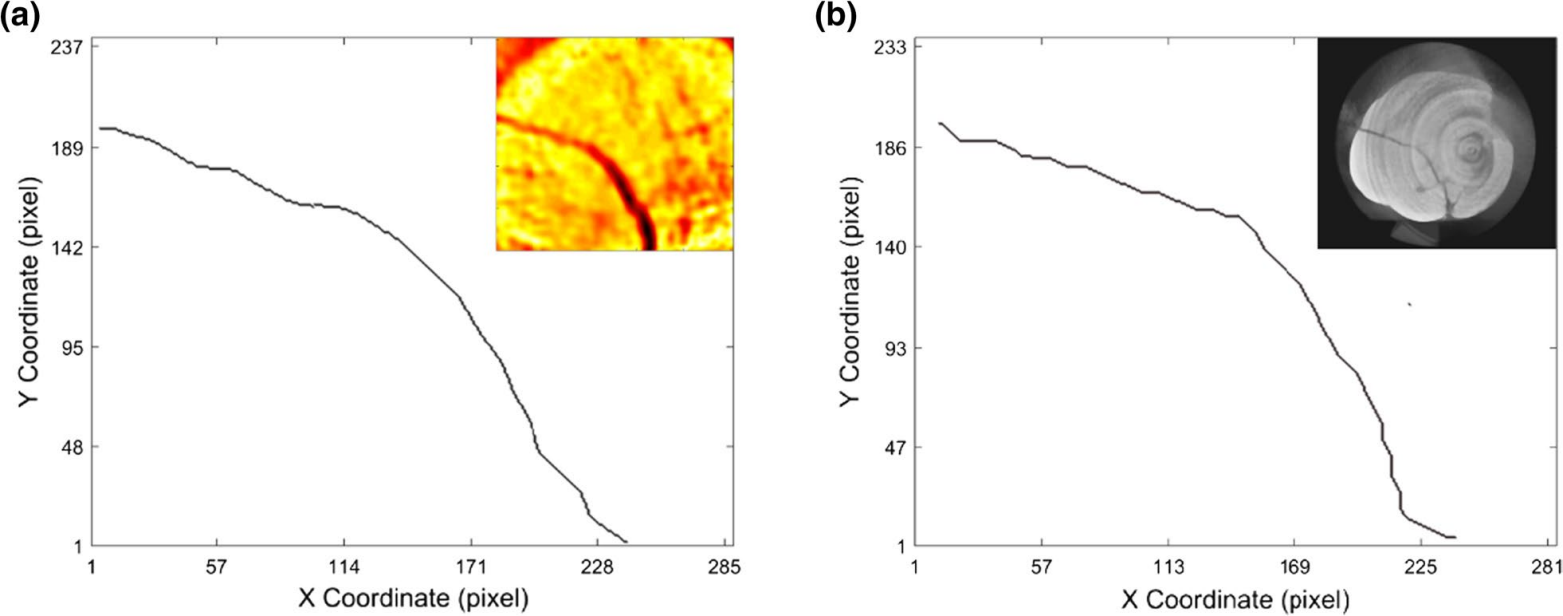
Skeletonization of the crack: **a** image from the principal strain field calculated by DIC; **b** skeleton from CT image. The inset was the original image for each case

In our case here, the principal strain field (Fig. 3e, f) was indicated to better detect the crack path compared with the displacement field or the unidirectional strain field (**ε**_**xx**_ or **ε**_**yy**_). The resulting principal strain field showed a high contrast with an emphasis on the crack region. The indicated crack area made a good agreement with the main crack captured from CT (the inset in Fig. 4b), which further demonstrated that the principal strain field was more appropriate for the crack detection method used here. It should be noted that several tiny cracks were seemed to be swallowed in the principal strain field, which was probably due to the relatively small deformation and the resolution of the strain calculation.

For cracked tooth diagnosis, the location of the crack was necessary for further dental treatment. As shown in Fig. 4a, b complete main crack was extracted and skeletonized as a binarized image based on the proposed iteration image treatment. Compared with the skeletonization of the micro-CT image (Fig. 4b), the main crack path obtained from the DIC strain field was in good agreement, which further proved the feasibility of the proposed method here. For the skeleton extraction, parameter like the lower threshold proposed in our case here may slightly influence the main crack skeletonization. The lower threshold needs to be adjusted to avoid the bifurcation of the main crack. The future work of the proposed image treatment should realize the adaptive setting of the thresholds based on statical analysis of the calculated strain field. Whereas, compared with the artificial neural network algorithm for crack extraction [36], the higher efficiency of the proposed methods here may be helpful for the development of the fast clinical crack tooth diagnosis.

Parameters such as the size of the subzone in the deformed image (sub-image) and the step size used in the displacement field calculation were crucial to the resolution of the resulting strain field. Small size of the sub-image would lead to fewer image features to be compared between the reference and deformed images, which would increase the matching error during the DIC process. Whereas, over size of the sub-image would cause an average effect for the details of the displacement field, which certainty decrease the resolution of the strain field. Another important parameter in DIC is the step size, which should be small enough to get the local displacement details. However, an undersized step-size will cause excessive noise, which would seriously affect the accuracy of extraction and positioning for the main crack here. Similar parameters for sub-image size and step size could also be found in reference [37].

### Imaging of crack opening displacement

**Fig. 5:**
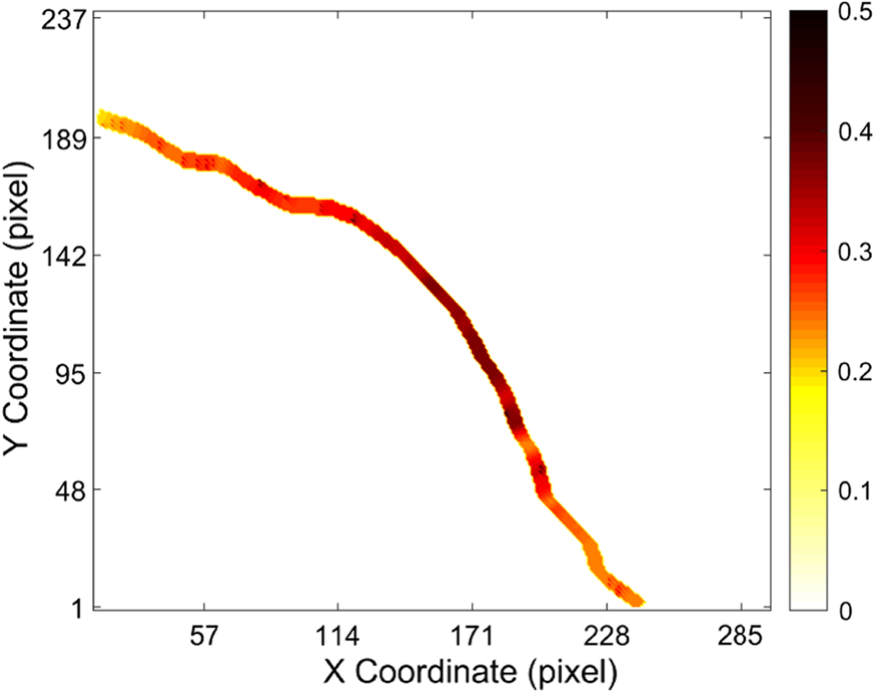
Visualization of the crack opening displacement. The unit of the value in the color bar was pixels. And 1 pixel responds to 20 µm

**Fig. 6:**
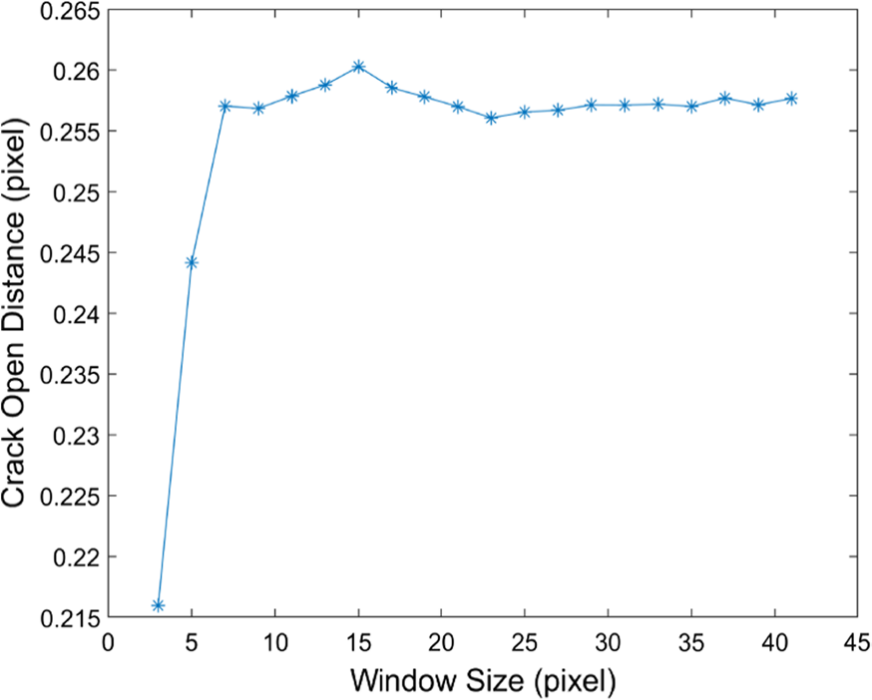
The calculated crack opening displacement varied with the parameter of the fitting window size used to calculate the inclination angle of local crack

The behavior of the crack can be evaluated and studied by measuring the displacement field around the crack. And the growth rate and direction of the crack are determined by several factors, such as the mechanical properties of the materials, load conditions, and structural geometry [38]. Quantitative characterization of the crack opening displacement may be helpful for the assessment of tooth damage, and it may also provide suggestions for dental repairment after then. As shown in Fig. 5, crack opening displacement was contoured with a color bar rendered in a heatmap mode. The crack opening displacement was found to be inhomogeneous, which ranged from 2 to 10 µm with a maximal value of 0.5 pixels (10 µm) located in the central region of the figure. The obtained magnitude of the COD was in good accordance with the magnitude of the crack width reported in the literature [10] in which the micro-crack in the cracked tooth was clinically found to be between 2 and 500 µm. It should be mentioned that the applied force onto the simulated cracked tooth was well controlled to be less than the force on the tooth during the normal mastication process. According to the work of Shinogaya et al. [39], the average forces on the mandibles during the mastication process were recorded between 1200 and 550 N. In our case here, the force applied to the simulated cracked tooth sample was carefully controlled as 500 N. And the encapsulated resin around the cracked tooth may also dissipate some energy, thus the force applied to the simulated cracked tooth should be considered as tiny and safe enough for the cracked tooth diagnosis. The obtained COD may be influenced by the parameter of the window size (ω) used for the calculation of the crack inclination angle. As shown in Fig. 6, the maximum calculated crack opening displacement initially increased with the window size (ω), and finally reached to a stable value. Here, the width of the fitting window was selected as 7 pixels, which make sure the calculated COD was stable converged.

For clinical diagnosis, considering the irregular geometry of human teeth, the proposed method may be performed in conjunction with dental operating microscope or some other customized microscopes. The local enlarged images were then used for the strain field calculation and crack extraction. Through linearization, it may be useful to reduce the measuring error caused by the 3D topography of the tooth. And it also should be pointed out that strain field calculation for 3D complex surface (like human tooth) may be one of the interesting and challenging research topics in further work.

### Error analysis

The uncertainty of the proposed image correlation system was evaluated by the random system error, which was measured based on 20 continuously static images. The mean value of the displacement for each image was calculated as:

14 $$g(x) = \frac{{\sum\nolimits_{i = 0}^{m} {\sum\nolimits_{j = 0}^{m} {\sqrt {{{u}}^{2} (x_{i} ,y_{j} ) + {{v}}^{2} (x_{i} ,y_{j} )} } } }}{m \times n}$$

where $g(x)$ is the average displacement, $m$ is the length of the image,$n$ is the width of the image, and $({x}_{i},{y}_{i})$ are the coordinates of the image, ***u ***is the displacement of the point $({x}_{i},{y}_{i})$ in X direction, and ***v*** is the displacement of the point $({x}_{i},{y}_{i})$ in Y direction.

**Fig. 7:**
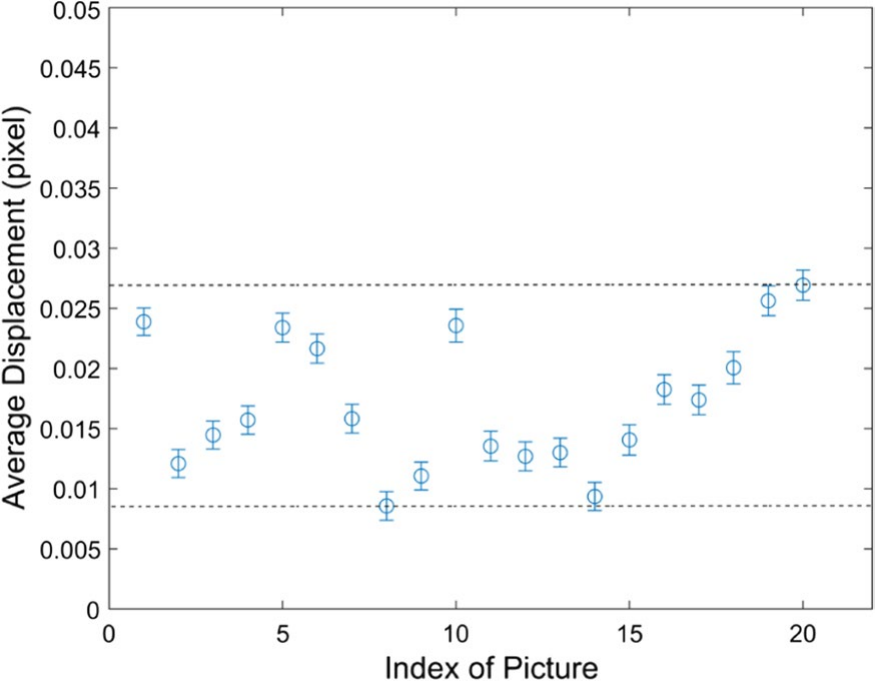
Random system error of the proposed image correlation system measured based on 20 continuously static images. Error bar means the standard deviation of the measured displacement field for each image

The ideal calculated displacement field for the static images should be found as zero, whereas the inevitable noise during the image acquisition process and the sub-region matching process would lead to certain error. As plotted in Fig. 7, the measured average displacement for each image randomly fluctuated between the maximum of 0.028 pixels (0.46 µm) and a minimum of 0.008 pixels (0.16 µm) with a variance of 0.001, which is two orders of magnitude lower than the value of the displacement field for the crack detection. Thus, the displacement measurement in the proposed crack detection should be considered valid and reliable.

## Conclusion

A method based on digital image correlation was established for crack detection of the simulated cracked tooth. The crack location can be extracted by skeletonization from the principal strain field. Compared with micro-CT, the detection of the main crack was in good accordance with results obtained from the proposed method. What’s more, the proposed method provides quantitatively visualization of the crack opening displacement. The measured crack opening displacement was found to be 2–10 µm under the normal mastication stress. The average displacement field resolution of the system built in this work is around 0.02 µm, which should have sufficient capability for crack tooth diagnosis. The proposed method provides an alternative solution for the clinical non-destructive portable detection of cracked teeth, and the results of the quantitative analysis may provide a guide for the evaluation of dental damage and the selection of subsequent dental restoration programs.

## Supplementary Information

**Additional file 1. Fig. S1.** Illustration of the calculation of the crack width based on displacement field obtained from digital image correlation.

## Data Availability

Not applicable.

## Abbreviations

DIC: Digital image correlation
COD: Crack opening displacement
