# Supplementary material for: A method of crack detection based on digital image correlation for simulated cracked tooth
Source: BMC Oral Health. 2021 Oct 19;21:539. doi: 10.1186/s12903-021-01897-2 (PMC8524926; doi:10.1186/s12903-021-01897-2)
Supplement: Supplementary file 1 — Additional file 1. Fig. S1. Illustration of the calculation of the crack width based on displacement field obtained from digital image correlation. [file 12903_2021_1897_MOESM1_ESM.docx]

**Additional file 1**

**A method of crack detection based on digital image correlation for simulated cracked tooth**

Chunliang Zhang^a^, Diwei Mo^a^, Juncheng Guo^a^, WenlongWang^a^*, Shangbin Long^a^, Houyao Zhu^a^, Danying Chen^b^, Guanghua Ge^c^, and Yadong Tang^d^*

*^a^School of Mechanical and Electrical Engineering, Guangzhou University, Guangzhou, 510006, China*

*^b^Hospital of Stomatology, Sun Yat-sen University; Guangdong Provincial Key Laboratory of Stomatology, Guangzhou, 510006, China*

*^c^Department of Dentistry, Hospital of Guangdong University of Technology, Guangdong University of Technology, Guangzhou, 510006, China*

*^d^School of Biomedical and Pharmaceutical Sciences, Guangdong University of Technology, Guangzhou, 510006, China*

Coresponding author: [wlwang@gzhu.edu.cn](mailto:wlwang@gzhu.edu.cn) (Wenlong WANG); [tangyadong@gdut.edu.cn](mailto:tangyadong@gdut.edu.cn) (Yadong TANG)

**
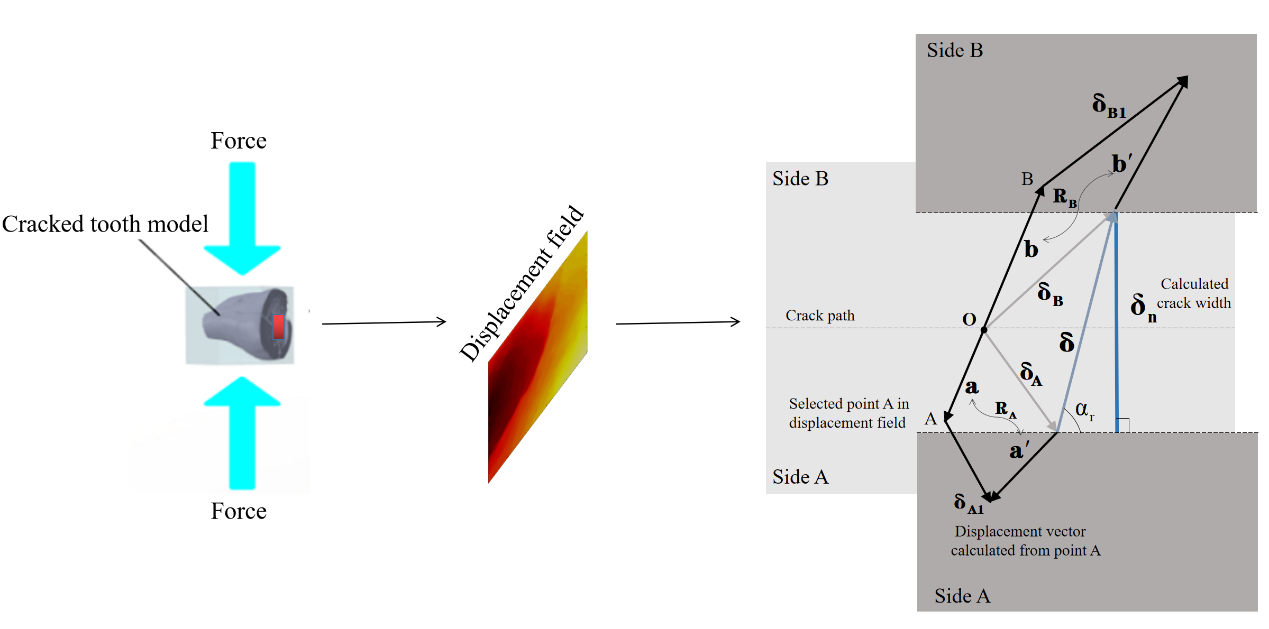
**

**Additional file 1: Fig. S1** Illustration of the calculation of the crack width based on displacement field obtained from digital image correlation.
